# Retraction Note: Numb inhibits epithelial-mesenchymal transition via RBP-Jκ-dependent Notch1/PTEN/FAK signaling pathway in tongue cancer

**DOI:** 10.1186/s12885-024-12253-2

**Published:** 2024-04-23

**Authors:** Jin-Yun Li, Wen-Xiao Huang, Xiao Zhou, Jie Chen, Zan Li

**Affiliations:** Department of Head and Neck Surgery, School of Medicine, Hunan Cancer Hospital and Affiliated Cancer Hospital of Xiangya, Central South University, No.283, Tongzipo Road, Yuelu District, 410013 Changsha, Hunan Province People’s Republic of China

**Retraction Note: BMC Cancer 19, 391 (2019)**

10.1186/s12885-019-5605-5

The Editors have retracted this article. After publication, concerns were raised regarding image similarities in the presented figures. Specifically:

- Figure 2b CAL27 NC and Numb 0h images appear highly similar;
- Figure 2b SCC-9 Numb 24h  and Fig. 5b SCC-9 RPB-Jk shRNA 0h images appear highly similar (with different brightness);
- Figure 2b SCC-9 NC 24h and Fig. 5b SCC-9 shNC 24h images appear highly similar (with different brightness);
- Figure 2b SCC-9 Numb 48h and Fig. 5b SCC-9 RPB-Jk shRNA 48h images appear highly similar (with different brightness);
- Figure 2d CAL27 Numb and Fig. 5d CAL27 RPB-Jk shRNA images appear highly similar (with rotation).

The authors have provided the raw data for validation. However, the Editors found some inconsistencies in the raw data, and determined them to be insufficient to address the above concerns. The Editors therefore no longer have confidence in the presented data.

The authors have not responded to any correspondence from the publisher about this retraction notice.

